# Predictive value of C-reactive protein to albumin ratio as a biomarker for initial and repeated intravenous immunoglobulin resistance in a large cohort of Kawasaki disease patients: a prospective cohort study

**DOI:** 10.1186/s12969-021-00517-1

**Published:** 2021-03-12

**Authors:** Xiaoliang Liu, Lin Wang, Kaiyu Zhou, Shuran Shao, Yimin Hua, Mei Wu, Lei Liu, Chuan Wang

**Affiliations:** 1 Department of Pediatric Cardiology, West China Second University Hospital, Sichuan University, No. 20, 3rd section, South Renmin Road, Chengdu, 610041 China; 2 Key Laboratory of Birth Defects and Related Diseases of Women and Children of Ministry of Education, West China Second University Hospital, Sichuan University, Chengdu, Sichuan China; 3 Key Laboratory of Development and Diseases of Women and Children of Sichuan Province, West China Second University Hospital, Sichuan University, Chengdu, Sichuan China; 4 Longquanyi District of Chengdu Maternity & Child Health Care Hospital, Chengdu, Sichuan China; 5 The Cardiac Development and Early Intervention Unit, West China Institute of Women and Children’s Health, West China Second University Hospital, Sichuan University, Chengdu, Sichuan China; 6 West China Medical School of Sichuan University, Chengdu, Sichuan China

**Keywords:** Kawasaki disease, C-reactive protein to albumin ratio, Intravenous immunoglobulin resistance

## Abstract

**Background:**

Intravenous immunoglobulin (IVIG) resistance prediction is one pivotal topic of interests in Kawasaki disease (KD). This study aimed to prospectively investigated the value of C-reactive protein-to-albumin (CAR) in predicting both initial and repeated IVIG resistance in patients with KD, and to test the hypothesis that CAR was more valuable or accurate than either C-reactive protein (CRP) or albumin (ALB) alone in IVIG resistance prediction.

**Method:**

A prospective cohort study involving 550 patients with KD was conducted. The clinical and laboratory data were compared between IVIG-response group and IVIG-resistance group. Multivariate logistic regression analysis was performed to identify the independent risk factors of initial/repeated IVIG resistance. Receiver operating characteristic (ROC) curves analysis was applied to assess the validity of CAR, CRP and ALB in predicting both initial and repeated IVIG resistance.

**Results:**

CAR was significantly higher in IVIG non-responders and was identified as independent risk factor for both initial and repeated IVIG resistance in KD. The best cut-off value of CAR for initial and repeated IVIG resistance prediction was 2.07 and 3.34, with a corresponding sensitivity of 0.610 and 0.548, a specificity of 0.552 and 0.813, respectively. The value of CAR was not better than either CRP or ALB alone for both initial and repeated IVIG resistance prediction.

**Conclusion:**

A higher CAR was an independent risk factor for both initial and repeated IVIG resistance. However, similar with that of CRP or ALB, the predictive value of CAR was not good enough for both initial and repeated IVIG resistance prediction in KD.

**Supplementary Information:**

The online version contains supplementary material available at 10.1186/s12969-021-00517-1.

## Background

Kawasaki disease (KD) is an acute systemic vasculitis with coronary artery lesions (CALs) as the most serious sequelae, predominantly affecting children younger than 5 years of age [1]. While timely initiation of therapy with intravenous immunoglobulin (IVIG) can effectively reduce the development of CALs [2], approximately 10–20% patients do not respond to initial IVIG treatment and have a higher risk of CALs [3]. For children suffering from initial IVIG resistance, repeated IVIG infusion (2 g/Kg given as a single intravenous infusion) is recommended by many experts despite there are currently no robust evidences from clinical trials to guide the clinicians in the choice of therapeutic agents [4–6]. However, approximately 5–10% of patients with KD are refractory to both initial and repeated IVIG therapy [7], and may benefit from adjunctive therapies for primary treatment, namely, corticosteroids [8, 9], infliximab [10, 11], plasma exchange [12, 13], cytotoxic agents [14, 15]. Thus, early prediction of both initial and repeated IVIG resistance is paramount in KD as those patients might improve from an early-intensified therapy.

Despite the etiology of KD remains unknown, it is well known that systematic inflammatory response plays a crucial role in pathogenesis of onset and progression of KD [16]. The innate immune system is activated as an early event of KD onset, evidenced by the activation of the interleukin (IL)-1, IL-6, and tumor necrosis factor (TNF) signaling pathways [16, 17]. Because of the association between inflammation and KD development, it is supposed that the concentrations of most acute-phase proteins (APRs), which are those whose plasma concentration increases (positive APRs) or decreases (negative APRs) by at least 25% during inflammatory disorders [18], could be used to measure the state of systematic inflammation response in KD.

Serum C-reactive protein (CRP) is a positive APR produced by hepatocytes upon activation by cytokines, such as IL-6 and TNF-a, and increased considerably with inflammation [19]. It was well established that the serum CRP was elevated in KD [20–23]. On the other hand, the albumin (ALB), which is traditionally regarded as a marker of nutritional status [24, 25], is also increasingly considered as the most important negative APR [18]. Catabolism of ALB is directly correlated with the severity of the acute inflammation. Hypoalbuminemia was commonly observed in patients with KD, which may be primarily resulted from the increasing permeability and leakage of serum ALB during the acute phase [24, 26]. Both serum CRP and ALB were commonly found to be associated with IVIG resistance and included in several risk-scoring systems for IVIG resistance prediction in KD [21–23, 27, 28]. However, both previous studies [29, 30] and ours [31] have documented that the predictive values of serum CRP and ALB as a single marker for IVIG resistance were not ideal. Notably, a newly introduced and novel parameter defined as the ratio of CRP to ALB (CAR), has been proposed as more valuable and accurate than either CRP or ALB alone in predicting inflammatory status and prognosis in various clinical settings, including the stable angina pectoris [32, 33], colorectal cancer [34, 35], coronary artery ectasia [36], inflammatory bowel disease [37], Takayasu arteritis [38], rheumatoid arthritis [39] and sepsis [40]. As a novel parameter, CAR might provide a variable capable of merging both information of CRP and ALB in patients with KD, nonetheless, the relationship of CAR and IVIG resistance in KD has never been evaluated.

Therefore, in the present study, we prospectively investigated the value of the newly defined CAR in predicting both initial and repeated IVIG resistance in patients with KD, and to test the hypothesis that CAR was more valuable or accurate than either CRP or ALB alone in IVIG resistance prediction.

## Patients and methods

Patients with KD were prospectively recruited between March 2015 and June 2019 at West China Second University Hospital of Sichuan University (WCSUH-SCU). The diagnosis of KD relies on standards recommended by American Heart Association (AHA) scientific statement for diagnosis, treatment, and long-term management of KD [1] and were confirmed by two experienced pediatricians (including ≥1 KD specialist). Structured questionnaires with pre-coded questions including basic demographic information, clinical manifestations, hematological examination results, treatment and follow-up outcomes, were used for data collection. All questionnaires were pretested and revised accordingly. Data collection was conducted by two well-trained doctors. The questionnaires were double-checked to assure its completeness. Informed written consent was obtained from parents after the nature of this study had been fully explained to them. The study was approved by the University Ethics Committee on Human Subjects at Sichuan University. All research was performed in accordance with relevant guidelines and regulations.

**Fig. 1:**
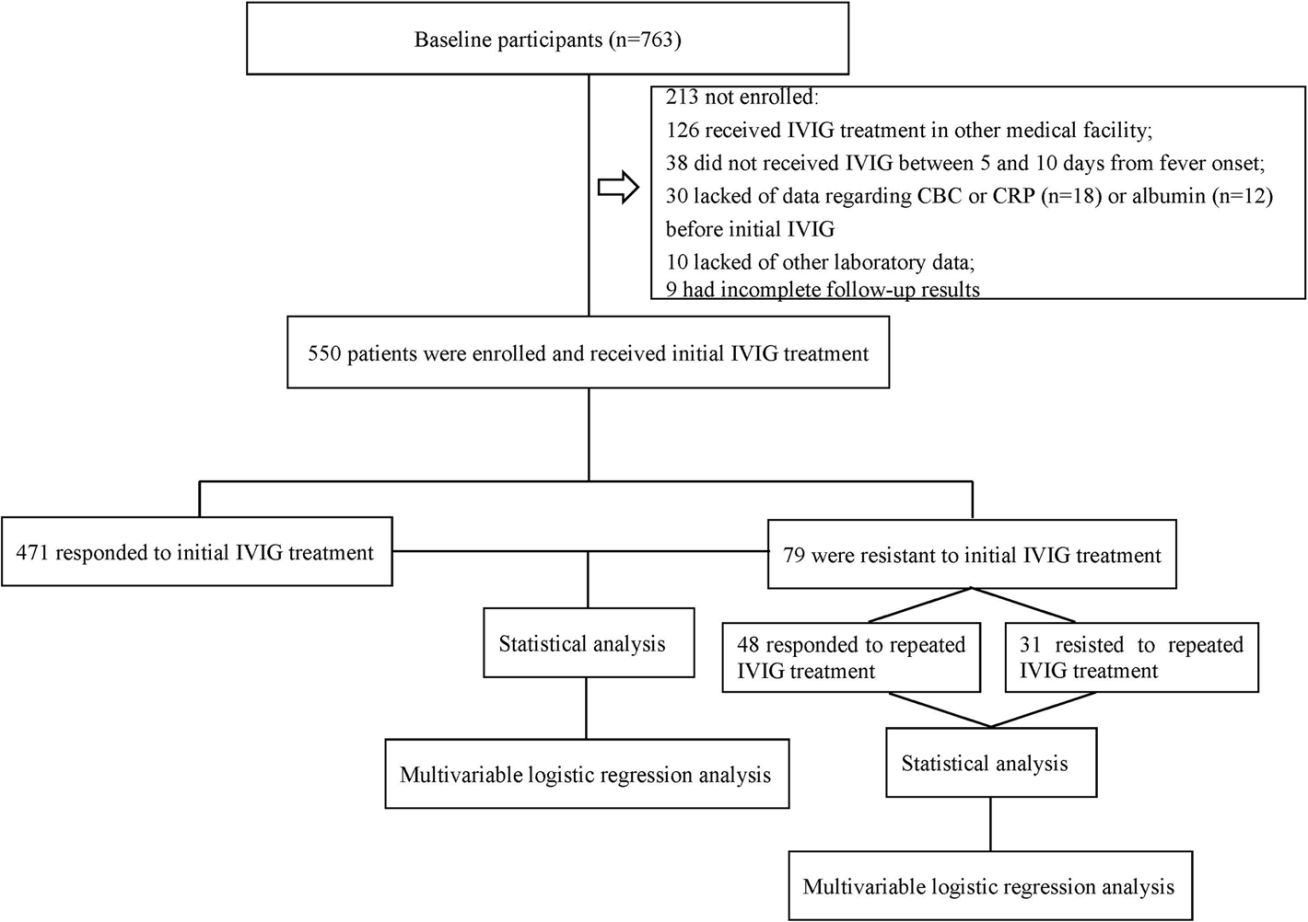
The flowchart of our prospective cohort study. In total, 763 patients were diagnosed with KD on admission. Those who had received IVIG treatment in other medical facilities (*n* = 126) or did not receive IVIG treatment before 10 days from fever onset (*n* = 38) were firstly excluded. Another 30 patients were also excluded due to lack of data regarding complete blood count (CBC) or CRP (*n* = 18) or serum ALB (*n* = 12) before initial IVIG. Additionally, we excluded 19 patients because other laboratory data (*n* = 10) or follow-up results (*n* = 9) were incomplete. Finally, 550 patients were enrolled for analysis, including 471 of initial IVIG responders and 79 of initial IVIG non-responders. Of the 79 patients with initial IVIG resistance, 31 of them did not respond to repeated IVIG treatment and received pulse intravenous methylprednisolone infusion

Since the serum ALB could be influenced by the nutritional status, patients with malnutrition and nutritional imbalance were firstly excluded using the screening tool for assessment of malnutrition in pediatrics (STAMP) [41]. After the exclusion, a total of 763 KD patients were enrolled. Thereafter, those who had received IVIG treatment in other medical facilities (*n* = 126) or did not receive IVIG treatment prior to 10 days from fever onset (*n* = 38) were excluded. Additionally, 30 patients were also excluded owing to lack of data regarding complete blood count (CBC) or CRP (*n* = 18) or serum ALB (*n* = 12) prior to initial IVIG. We also excluded 19 patients because other laboratory data (*n* = 10) or follow-up results (*n* = 9) were incomplete. Finally, 550 patients were enrolled for analysis, including 471 initial IVIG responders and 79 initial IVIG non-responders. Of the 79 patients with initial IVIG resistance, 31 did not respond to repeated IVIG treatment and received pulse intravenous methylprednisolone infusion (Fig. 1). No patients received additional treatment such as infliximab, plasma exchange and cytotoxic agents. The CAR was calculated by dividing CRP by ALB collected before initial IVIG both for initial and repeated IVIG resistance. In case of more than one CRP or albumin determination before the initial IVIG infusion, the highest value of CRP and the lowest value of albumin were chosen for the analysis.

All patients received the same standard treatment regimen of KD. Aspirin (30–50 mg/kg/day) and IVIG (2 g/kg given as a single intravenous infusion) were administered within the first 10 days of illness from fever onset. After patients defervesce for 48–72 h, a tapered dose of aspirin (3–5 mg/kg/day) was administered for 6–8 weeks. If patients had CALs, aspirin was continued until there was no evidence of CALs. If the patient had initial IVIG resistance, which was defined as recurrent or persistent fever or other clinical signs of KD for at least 36 h but not longer than 7 days after initial IVIG [42], the second IVIG (2 g/kg given as a single intravenous infusion) was administered. Furthermore, if the patient had recurrent or persistent fever after the second IVIG infusion, which was defined as the repeated IVIG resistance, tapered administration of pulse intravenous methylprednisolone (20–30 mg/kg/day for 3 consecutive days) followed by oral prednisone (2 mg/kg/day) for 7 days were given as adjunctive therapy.

CALs were defined on the normalization of dimensions for body surface area (BSA) as Z scores (standard deviation units from the mean, normalized for BSA) as follows: no involvement (z score < 2.0), dilation (z score ≥ 2.0 to < 2.5), aneurysm (z ≥ 2.5; z ≥ 10 for giant aneurysm) of coronary arteries on the basis of maximal internal diameters of the right coronary artery (RCA), left anterior descending artery (LAD) and left circumflex coronary artery (LCX) [1, 43]. According to institutional protocol, patients underwent standardized echocardiograms by two pediatric ultrasonologists during the acute phase and 6–8 weeks later in cardiology clinic follow-up evaluations, until the resolution of CALs. Body surface area and z scores were calculated using the Haycock [44] and the Kobayashi equations [45], respectively.

## Statistical analyses

Data analysis was performed with SPSS 21.0 (IBM SPSS Statistics version 21.0, Armonk, NY, IBM Corp.). Quantitative data are presented as the median with the 25th and 75th percentiles (interquartile range (IQR)) in square brackets, while qualitative data are expressed as n/% as appropriate. Shapiro-Wilk test and homogeneity test of variance were used to confirm that quantitative data from different groups come from a normal distribution and meet the homogeneity of variance. The chi-square test and unpaired Student’s t test/Mann-Whitney U test were applied to compare demographic characteristics, clinical manifestations, and laboratory data between groups.

Numerical variables that showed statistical significance in the univariate analysis were transformed into dichotomous variables. Cut-off values, located in the maximum value of the Youden Index according to sensitivity and specificity, were selected on the basis of the receiver operating characteristic (ROC) curve. These crucial indicators from univariate analysis were then subjected to multivariate logistic regression analysis to identify independent predictors of IVIG resistance. Odds ratio (OR) values were used to determine the score of each independent risk factor and construct a prediction model. The best cut-off points of the multivariable model for IVIG resistance prediction and its corresponding predictive power were further assessed by the ROC curve. It was considered statistically significant when *P* values were < 0.05.

To compare the predictive value of CAR and ALB or CRP for IVIG resistance, ROC analysis was conducted to determine the best cut-off values and their corresponding predictive validities. Sensitivity, specificity, positive predictive value (PPV), negative predictive value (NPV), and diagnostic accuracy were assessed. Additionally, two multivariable models for IVIG resistance prediction with either CAR or CRP and ALB were constructed. De Long test was used to compare ROC curves.

## Results

### Comparison of subjects between groups of initial IVIG-response and IVIG-resistance

As seen in Table 1, there was no significant difference between the two groups regarding gender proportion, body mass index (BMI) and fever duration before initial IVIG treatment, as well as typical clinical manifestations of KD (rash, extremity changes, conjunctivitis, oral changes; *p* > 0.05). The incidence of CALs was relatively higher in the initial IVIG-resistance group but did not reach statistical significance (13.9% vs 8.9%, *p* = 0.153). When compared with patients from the initial IVIG-response group, patients from initial IVIG-resistance group were older, presenting a higher incidence of cervical lymphadenopathy with substantially higher levels of neutrophil-lymphocyte ratio (NLR), platelet-lymphocyte ratio (PLR), and total bilirubin (TBil), but lower levels of serum sodium (Na^+^) (*p* < 0.005).

**Table 1:** Comparison of clinical data between the groups of initial IVIG-response and IVIG-resistance in KD

	IVIG-responsive (***n*** = 471)	IVIG-resistance (***n*** = 79)	***p*** value
Male	271 (57.5)	43 (54.4)	0.625
Age, months	24.0 (13.0–42.0)	28.0 (14.0–54.0)	0.016
BMI, kg/m^2^	15.3 (14.5–16.0)	15.1 (14.3–16.2)	0.487
**Clinical manifestations**			
Rash	365 (77.5)	67 (84.8)	0.182
Extremity changes	286 (60.7)	41 (51.9)	0.173
Conjunctivitis	431 (91.5)	71 (89.9)	0.666
Oral changes	599 (91.0)	99 (94.3)	0.347
Cervical lymphadenopathy	193 (41.0)	42 (53.2)	0.049
Fever duration before initial IVIG, days	5.0 (5.0–6.0)	5.0 (5.0–6.0)	0.313
Incomplete KD	173 (36.7)	25 (31.6)	0.054
CALs	41/463 (8.9)	11/79 (13.9)	0.153
**Before initial IVIG**			
WBC, ×10^9^/L	13.3 (10.6–16.8)	14.3 (10.6–17.5)	0.718
Hemoglobin, g/L	109.0 (101.0–116.0)	107.0 (99.0–114.0)	0.346
CRP, mg/L	70.0 (41.0–106.0)	90.0 (62.0–144.0)	0.001
ESR, mm/h	64.0 (46.0–81.0)	67.0 (47.0–96.0)	0.242
AST, U/L	31.0 (25.0–48.0)	34.0 (25.0–65.0)	0.485
ALT, U/L	35.0 (21.0–78.0)	53.0 (27.0–120.0)	0.085
ALB, g/L	38.0 (35.0–41.0)	36.0 (32.0–39.0)	<0.001
TBil, μmol/L	6.0 (4.0–8.0)	7.0 (4.0–15.0)	<0.001
Na^+^, mmol/L	137.0 (135.0–139.0)	135.0 (133.0–137.0)	<0.001
NLR	2.63 (1.71–4.80)	4.83 (2.92–8.65)	<0.001
PLR	103.9 (74.8–151.1)	137.2 (93.9–235.0)	<0.001
CAR	1.86 (1.04–2.88)	2.60 (1.67–4.27)	<0.001

Abbreviations: *ALB* Albumin; *AST* Aspartate aminotransferase; *ALT* Alanine aminotransferase; *BMI* Body mass index; *CRP* C-reactive protein; *CAR* C-reactive protein-to-albumin ratio; *CALs* Coronary artery lesions; *ESR* Erythrocyte sedimentation rate; *IVIG* Intravenous immunoglobulin; *KD* Kawasaki disease; *NLR* Neutrophil-lymphocyte ratio; *Na*^*+*^ Sodium; *PLR* Platelet-lymphocyte ratio; *TBil* Total bilirubin; *WBC* White blood cell;

The data are presented as the median with the 25th and 75th percentiles in square brackets for continuous variables and as the percentage for the categorical variables

In KD patients presenting with initial IVIG resistance, parameters of CRP (90.0 mg/L[IQR:62.0–144.0] vs 70.0 mg/L [41.0–106.0], *p* = 0.001) and CAR (2.60[1.67–4.27] vs 1.86[1.04–2.88], *p <* 0.001) were significantly higher than initial IVIG responders, whereas ALB (36.0 g/L [32.0–39.0] vs 38.0 g/L [35.0–41.0], *p* < 0.001) of initial IVIG non-responders were significantly decreased.

### Predictive model 1 construction with CAR for initial IVIG resistance

Statistically significant variables including age, cervical lymphadenopathy, NLR, PLR, TBil, Na^+^, and CAR from the univariate analysis were enrolled in the multivariate logistic regression analysis. It was identified that CAR ≥2.07, TBil ≥9.5 μmol/L and Na^+^ ≤ 135.7 mmol/L were independent risk factors for initial IVIG resistance. These results are depicted in Table 2.

**Table 2:** A multivariate logistic regression analysis for predicting initial IVIG resistance in KD

Model 1	***β***	SE	Walds	***P*** value	OR	95% CI	Points
Age ≥ 48 months	0.000	0.006	0.001	0.976	1.000	0.989–1.011	
Cervical lymphadenopathy	−0.245	0.272	0.815	0.367	0.782	0.459–1.333	
NLR ≥ 2.91	0.584	0.354	2.720	0.099	1.794	0.896–3.592	
PLR ≥ 134	0.260	0.303	0.736	0.391	1.297	0.716–2.351	
CAR≥2.07	0.762	0.321	5.629	0.018	2.142	1.142–4.019	5.0
TBil≥9.5 μmol/L	0.566	0.282	4.040	0.044	1.761	1.014–3.058	3.5
Na^+^ ≤ 135.7 mmol/L	−0.729	0.270	7.322	0.007	0.482	0.284–0.818	4.5
Intercept	1.214	0.425	8.174	0.004	3.366	–	
**Model 2**							
Age ≥ 48 months	0.425	0.317	1.793	0.181	1.529	0.821–2.848	
Cervical lymphadenopathy	−0.237	0.272	0.758	0.384	0.789	0.463–1.345	
NLR ≥ 2.91	0.452	0.354	1.628	0.202	1.571	0.785–3.146	
PLR ≥ 134	0.249	0.305	0.669	0.413	1.283	0.706–2.331	
CRP ≥ 57.7 mg/L	0.567	0.324	3.062	0.080	1.762	0.934–3.325	
ALB≤34.1 g/L	−0.698	0.276	6.398	0.011	0.497	0.290–0.855	5.0
TBil≥9.5 μmol/L	0.589	0.287	4.227	0.040	1.803	1.028–3.162	4.0
Na^+^ ≤ 135.7 mmol/L	−0.670	0.274	6.007	0.014	0.512	0.299–0.874	4.5
Intercept	1.160	0.441	6.931	0.008	3.191	–	

Abbreviations: *ALB* Albumin; *CRP* C-reactive protein; *CAR* C-reactive protein-to-albumin ratio; *IVIG* Intravenous immunoglobulin; *KD* Kawasaki disease; *NLR* Neutrophil-lymphocyte ratio; *Na*^*+*^ Sodium; *PLR* Platelet-lymphocyte ratio; *TBil* Total bilirubin;

The parameters of CAR ≥2.07, TBil ≥9.5 μmol/L and Na^+^ ≤ 135.7 mmol/L were incorporated into the predictive model 1 for initial IVIG resistance. On the basis of OR values, score points for each variable were as follows: CAR, 5 points; TBil, 3.5 points; Na^+^, 4.5 points. The best cutoff point for high risk initial IVIG resistance was ≥5.0 points, with an area under the curve (AUC) of 0.599 (95% CI, 0.556–0.640, *p* < 0.001), sensitivity of 0.670 and specificity of 0.527.

### Predictive model 2 construction with CRP and ALB for initial IVIG resistance

Statistically significant variables including age, cervical lymphadenopathy, NLR, PLR, CRP, TBil, Na^+^, and ALB were enrolled in multivariate logistic regression analysis. It was identified that ALB ≤34.1 g/L, TBil ≥9.5 μmol/L and Na^+^ ≤ 135.7 mmol/L were independent risk factors for initial IVIG resistance. These results are depicted in Table 2.

The parameters of ALB ≤34.1 g/L, TBil ≥9.5 μmol/L and Na^+^ ≤ 135.7 mmol/L were incorporated into predictive model 2 for initial IVIG resistance. On the basis of OR values, score points for each variable were as follows: ALB, 5.0 points; TBil, 4.0 points; Na^+^, 4.5 points. The discriminated cutoff point for high risk initial IVIG resistance was ≥5.0 points, with an AUC of 0.642 (95% CI, 0.600–0.682, *p* < 0.001), sensitivity of 0.544 and specificity of 0.739.

In predicting initial IVIG resistance, models 1 and 2 yielded a similar predictive ability without significant statistical difference (*p* = 0.170) (Supplementary material 1).

### Predictive ability of CAR, CRP, and ALB in predicting initial IVIG resistance

**Fig. 2:**
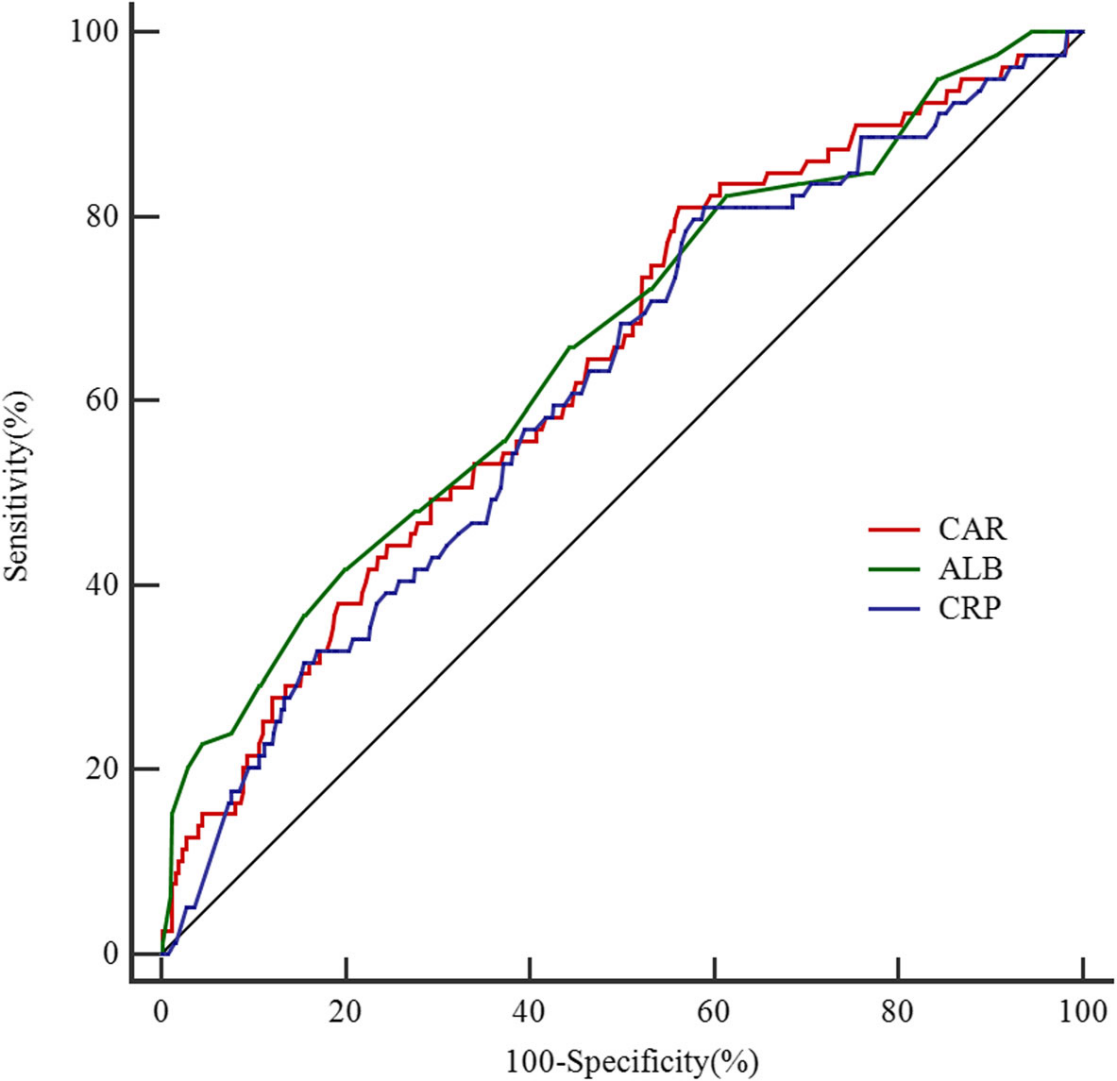
The receiver operating characteristic (ROC) curve for CAR, C-reactive protein and serum albumin in predicting initial IVIG resistance

The parameter of CAR ≥2.07, CRP ≥ 57.7 mg/L, and ALB ≤34.1 g/L produced a sensitivity, specificity, PPV, and NPV of 0.610, 0.552, 0.185, 0.894; 0.810, 0.410, 0.187, 0.928 and 0.418, 0.800, 0.260, 0.891, respectively. The AUC value of CAR (AUC: 0.639, 95%CI: 0.597–0.679) was significantly higher than that of CRP (AUC: 0.616, 95%CI: 0.574–0.657) (*p* = 0.002), but not significantly different from ALB (AUC: 0.657, 95%CI: 0.616–0.697) (*p* = 0.633) (Fig. 2). Additionally, diagnostic sensitivity and specificity of CAR, CRP and ALB according to the ROC optimized decision limits in predicting initial IVIG resistance are shown in Supplementary material 2.

### Comparison of subjects between repeated IVIG response and IVIG resistance group

A total of 79 patients with KD were identified as initial IVIG resistant and received repeated IVIG treatment. Comparison of clinical data between repeated IVIG response (*n* = 48) and resistance (*n* = 31) were shown in Table 3. Age, fever duration prior to initial IVIG infusion, gender proportion, BMI, typical clinical features, incidence of incomplete KD and CALs were not significantly different between groups (*p* > 0.05). When compared with patients from the repeated IVIG-response group, patients from the repeated IVIG-resistance group had significantly higher levels of TBil and NLR but lower levels of Na^+^ (*p* < 0.05). In KD patients presenting repeated IVIG resistance, parameters of CRP (122.0 mg/L [72.0–168.0] vs 79.0 mg/L [58.5–109.5], *p* = 0.006) and CAR (3.68[2.11–4.76] vs 2.02[1.62–3.16], *p* = 0.002) were significantly higher than repeated IVIG responders, while ALB (33.0 g/L [29.0–38.0] vs 37.0 g/L [33.0–39.8], *p* = 0.010) of repeated IVIG non-responders significantly decreased.

**Table 3:** Comparison of clinical data between the groups of repeated IVIG responders and non-responders in KD

	IVIG-responsive (***n*** = 48)	IVIG-resistance (***n*** = 31)	***p*** value
Male	25 (52.1)	18 (58.1)	0.649
Age, months	28.0 (13.0–55.0)	29.0 (14.0–54.0)	0.885
BMI, kg/m^2^	15.0 (14.1–15.9)	15.3 (14.5–16.1)	0.539
**Clinical manifestations**			
Rash	40 (83.3)	30 (96.8)	0.141
Extremity changes	26 (54.2)	15 (48.4)	0.651
Conjunctivitis	43 (89.6)	28 (90.3)	1.000
Oral changes	45 (93.8)	31 (100.0)	0.276
Cervical lymphadenopathy	26 (54.2)	16 (51.6)	1.000
Fever duration before initial IVIG, days	5.0 (5.0–7.0)	5.0 (5.0–6.0)	0.123
Incomplete KD	17 (35.4)	8 (25.8)	0.461
CALs	7 (14.6)	4 (12.9)	1.000
**Before initial IVIG**			
WBC, ×10^9^/L	14.5 (11.0–17.5)	13.9 (9.0–20.1)	0.814
Hemoglobin, g/L	109.0 (102.0–115.8)	104.0 (96.0–111.0)	0.161
CRP, mg/L	79.0 (58.5–109.5)	122.0 (72.0–168.0)	0.006
ESR, mm/h	67.0 (47.5–95.0)	67.0 (40.0–100.0)	0.917
AST, U/L	35.0 (24.3–75.3)	34.0 (28.0–57.0)	0.204
ALT, U/L	57.0 (23.0–133.0)	46.0 (34.0–92.0)	0.173
ALB, g/L	37.0 (33.0–39.8)	33.0 (29.0–38.0)	0.010
TBil, μmol/L	7.0 (5.0–13.0)	7.0 (4.0–42.0)	0.044
Na^+^, mmol/L	136.0 (134.0–138.0)	134.0 (131.0–135.0)	0.007
NLR	4.12 (2.93–7.66)	7.67 (2.52–12.27)	0.020
PLR	136.8 (92.4–214.4)	146.4 (93.9–329.9)	0.052
CAR	2.02 (1.62–3.16)	3.n (2.11–4.76)	0.002

Abbreviations: *ALB* Albumin; *AST* Aspartate aminotransferase; *ALT* Alanine aminotransferase; *BMI* Body mass index; *CRP* C-reactive protein; *CAR* C-reactive protein-to-albumin ratio; *CALs* Coronary artery lesions; *ESR* Erythrocyte sedimentation rate; *IVIG* Intravenous immunoglobulin; *KD* Kawasaki disease; *NLR* Neutrophil-lymphocyte ratio; *PLR* Platelet-lymphocyte ratio; *TBil* Total bilirubin; *Na+* Sodium; *WBC* White blood cell;

The data are presented as the median with the 25th and 75th percentiles in square brackets for continuous variables and as the percentage for the categorical variables

### Predictive model 1 construction with CAR for repeated IVIG resistance

Statistically significant variables including CAR, TBil, Na^+^, and NLR from the univariate analysis were enrolled in the multivariate logistic regression analysis. It was identified that CAR ≥3.34, TBil ≥41.0 μmol/L, and Na^+^ ≤ 135.0 mmol/L were independent risk factors for repeated IVIG resistance and results were depicted in Table 4.

**Table 4:** A multivariate logistic regression analysis for predicting repeated IVIG non-responders in KD

Model 1	***β***	SE	Walds	***P*** value	OR	95% CI	Points
CAR≥3.34	1.472	0.594	6.152	0.013	4.359	1.362–13.950	3.5
TBil≥41 μmol/L	2.027	0.912	4.939	0.026	7.589	1.270–45.332	5.0
Na^+^ ≤ 135 mmol/L	−1.276	0.564	5.119	0.024	0.279	0.092–0.843	3.0
NLR ≥ 6.86	0.394	0.592	0.443	0.505	1.483	0.465–4.734	
Intercept	−1.894	0.988	3.676	0.055	0.150	–	
**Model 2**							
CRP ≥ 102.0 mg/L	0.958	0.585	2.685	0.101	2.606	0.829–8.193	
ALB≤34.0 g/L	−0.822	0.563	2.128	0.145	0.440	0.146–1.326	
TBil≥41 μmol/L	2.037	0.943	4.662	0.031	7.666	1.207–48.696	5.0
Na^+^ ≤ 135 mmol/L	−1.288	0.558	5.323	0.021	0.276	0.092–0.824	3.0
NLR ≥ 6.86	0.279	0.602	0.215	0.643	1.322	0.406–4.306	
Intercept	−1.060	1.030	1.058	0.304	0.347	–	

Abbreviations: *ALB* Albumin; *CRP* C-reactive protein; *CAR* C-reactive protein-to-albumin ratio; *IVIG* Intravenous immunoglobulin; *KD* Kawasaki disease; *NLR* Neutrophil-lymphocyte ratio; *PLR* Platelet-lymphocyte ratio; *TBil* Total bilirubin; *Na*^*+*^ Sodium;

The parameters of CAR ≥3.34, TBil ≥41.0 μmol/L, and Na^+^ ≤ 135.0 mmol/L were incorporated into predictive model 1 for repeated IVIG non-responders. On the basis of OR values, score points for each variable were as follows: CAR, 3.5 points; TBil, 5.0 points; Na^+^, 3.0 points. The best cutoff point for high risk repeated IVIG resistance was ≥3.5 points, with an AUC of 0.708 (95% CI, 0.595–0.805, *p* < 0.001), sensitivity of 0.516 and specificity of 0.875.

### Predictive model 2 construction with CRP and ALB for repeated IVIG resistance

Statistically significant variables including CRP, ALB, TBil, Na^+^, and NLR from the univariate analysis were enrolled in the multivariate logistic regression analysis. It was identified that TBil ≥41.0 μmol/L and Na^+^ ≤ 135.0 mmol/L were independent risk factors for repeated IVIG resistance. These results were depicted in Table 4.

The parameters of TBil ≥41.0 μmol/L and Na^+^ ≤ 135.0 mmol/L were incorporated into predictive model 2 for repeated IVIG resistance. On the basis of OR values, score points for each variable were as follows: TBil, 5.0 points; Na^+^, 3.0 points. The discriminated cutoff point for high risk repeated IVIG non-responders was ≥3.0 points, with an AUC of 0.740 (95%CI, 0.629–0.832, *p* < 0.001), sensitivity of 0.774 and specificity of 0.646.

In predicting repeated IVIG resistance, model 1 presented similar predictive ability to model 2, without a significant statistical difference (*p* = 0.629) (Supplementary material 3).

### Predictive ability of CAR, CRP, and ALB in predicting repeated IVIG resistance

**Fig. 3:**
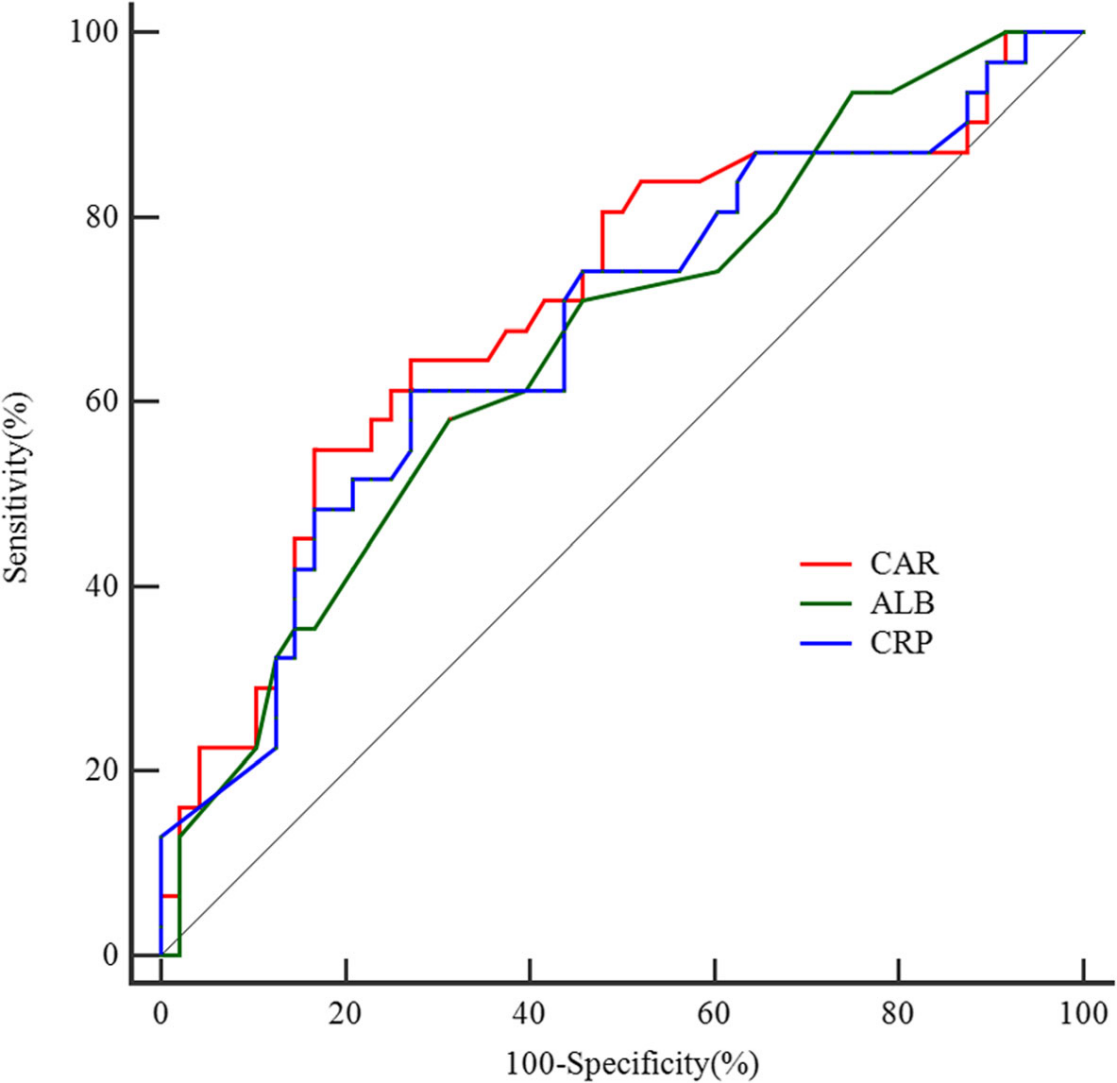
The receiver operating characteristic (ROC) curve for CAR, C-reactive protein and serum albumin in predicting repeated IVIG resistance

The parameters of CAR ≥3.34, CRP ≥ 102.0 mg/L, and ALB ≤34.0 g/L produced a sensitivity, specificity, PPV, NPV of 0.548, 0.813, 0.654, 0.741; 0.613, 0.729, 0.594, 0.745 and 0.581, 0.688, 0.546, 0.717, respectively. The AUC value of CAR (AUC: 0.703, 95% CI: 0.590–0.801) was similar to that of CRP (AUC: 0.674, 95%CI: 0.560–0.776) (*p* = 0.094) and ALB (AUC: 0.664, 95%CI: 0.549–0.767) (*p* = 0.585) (Fig. 3). Additionally, diagnostic sensitivity and specificity of CAR, CRP, and ALB according to the ROC optimized decision limits in predicting repeated IVIG resistance are shown in Supplementary material 4.

### The validity of CAR in predicting initial and repeated IVIG resistance for the normal and abnormal ALT groups

For the initial IVIG resistance prediction, the patients were divided into the normal alanine aminotransferase (ALT) group (*n* = 335) and increased ALT group (*n* = 215) using the cutoff of 40 U/L [46]. It showed significant difference for the normal group (1.66[0.90–2.70] vs 2.28[1.35–3.33], *p* = 0.005), while borderline significant difference was evidenced in CAR between the IVIG responders and non-responders for the increased ALT group (1.86[1.04–2.88] vs 2.60[1.52–4.29], *p* = 0.05). The best cutoff value of CAR for predicting initial IVIG resistance in normal and increased ALT group were 2.29 and 2.47, yielding a sensitivity, specificity, PPV, NPV of 0.55, 0.68, 0.18, 0.92 and 0.54, 0.57, 0.23, 0.84, respectively, the predictive ability of which were not much better than that of CAR≥2.07 in the total group.

For repeated IVIG resistance prediction, patients were divided into normal ALT group (*n* = 38) and increased ALT group (*n* = 41) accordingly. There were significant differences in CAR between repeated IVIG responders and non-responders for the normal ALT group (1.75[1.40–3.28] vs 3.60[1.99–5.09], *p* = 0.045) and the increased ALT group (2.18[1.67–3.00] vs 3.92 [2.10–4.84], *p* = 0.013). The best cutoff value of CAR for predicting repeated IVIG resistance in normal and increased ALT group were 2.7 and 2.91, which did not enhance the predictive value with lower specificity of 0.62 and 0.70, respectively, despite the sensitivity (0.65 and 0.71) was slightly elevated.

## Discussion

Initial IVIG resistance prediction is one of the primary clinical issues and study hotspots in KD. In the acute phase of KD, it has been found that the level of CRP reflecting systemic inflammatory burden increases while serum ALB decreases for its increasing permeability and leakage due to vascular inflammation [24, 26]. Both parameters associated with IVIG resistance and were enrolled in several risk-scoring systems for initial IVIG resistance prediction in KD [20–23, 27, 28, 47]. However, both previous studies [29, 30] and ours [31] documented that the predictive values of serum CRP and ALB as a single marker for initial IVIG resistance were not ideal. Notably, CAR as a ratio of CRP to ALB initially proposed by Fairclough [48], has been widely demonstrated to be more valuable and accurate than either CRP or ALB alone, providing a variable capable of merging information in the prediction of the systemic inflammatory state and prognosis in adverse cardiovascular events, cancers, inflammatory bowel disease, arteritis and critically ill septic patients [32–40, 48–51]. In almost previous studies, CAR performed better predictive ability than either CRP or ALB alone, nonetheless, Cagdas et al. found that CAR had better predictive accuracy than CRP but was not superior to ALB [52]. Thus, our findings agreed with the latter, that a higher CAR was better than CRP and similar with ALB in predicting initial IVIG resistance. The “unexpected” outcome might be attributed to the asynchronous pathogenesis process and change in time of CRP and hypoalbuminemia during the acute process of KD. It was found that peak time of serum CRP and ALB level was not consistent, which was 36–48 h and about 5 days after onset of inflammation stimulation, respectively [18]. The median time of blood test in the present study was 5 days from fever onset before initial IVIG infusion, which was almost consistent with the peak concentration time of ALB but passed that of CRP. In addition, CAR and ALB were identified as the independent risk factors for initial IVIG resistance, while CRP was not. Therefore, compared to CRP, CAR and ALB in the present study were more likely to reflect the inflammatory status of patients with KD. On the basis of these evidences, we speculated that our findings could possibly and partly explained by the different kinetics of CRP and ALB in inflammatory state. However, other underlying involved mechanisms also warrant to be further clarified.

There is a paucity of data concerning repeated IVIG resistance, and the role of CAR, in this regard, has not been investigated. Previous clinical trials suggested that adding corticosteroid [53, 54] or cyclosporine [55] agents to the standard treatment regimen of KD could reduce the rate of initial IVIG resistance and further decrease the incidence of CALs among high-risk patients with KD for initial IVIG resistance predicted by risk-scoring systems in Japan [21–23], whereas, approximately 10–20% were still resistant to initial IVIG treatment [56]. Aforementioned findings suggested the repeated IVIG resistance prediction was also essential and clinically significant in that patients with KD, at a high risk of repeated IVIG resistance, might benefit from more aggressive therapy. Here, we found that repeated IVIG non-responders presented with a remarkably higher CAR level than responders, indicating some extent of residual inflammation. A best cutoff value of 3.34 for repeated non-responders yielded a higher specificity of 0.813, but moderate sensitivity of 0.548, PPV of 0.654 and NPV of 0.741. Although all non-responders for repeated IVIG resistance could not be identified by CAR, these findings, nonetheless, may expand the limited information regarding repeated IVIG resistance prediction and provide some references for clinical management.

Additionally, the prediction of CALs is of equal clinical importance as IVIG resistance. However, CAR did not differentiate between CALs and non-CALs in patients with KD (1.94[1.27–3.32] vs 1.92[1.08–2.97], *p* = 0.283). Previous findings suggested that a persistent and ongoing inflammatory reaction might be more likely associated with the development of CALs. In comparison with our baseline CAR, its fluctuation might possess greater predictive power for CALs in patients with KD. Therefore, further study might collect different time points of CAR to elevate its predictive ability and prognosis of CALs.

This study must be viewed in light of some potential limitations. Firstly, selective bias may occur as this study was performed in a single institution. Secondly, the findings might be only applicable to KD patients receiving the standardized IVIG treatment (2 g/Kg) prior to 10 days from fever onset. Despite the above limitations, this study is the first to determine the predictive value of CAR for both initial and repeated IVIG resistance with a large sample size and prospective approach. It was identified that CAR was significantly higher in patients with IVIG resistance and was an independent risk factor for both initial and repeated IVIG resistance, but may serve as a complementary laboratory marker for IVIG resistance prediction in KD. Disproving our hypothesis, it was found that a higher CAR was better than CRP and similar to ALB in predicting initial IVIG resistance, whereas its predictive ability for repeated IVIG resistance was also similar to ALB and CRP. Additionally, the predictive models 1 (with CAR) and 2 (with Albumin and CRP) were not statistically different in prediction of both initial and repeated IVIG resistance. It was nothing less but definitely nothing more. Due to an unknown origin of KD and in light of the above findings, we speculate a prediction model combined with other specific indicators rather than clinical and routine laboratory variables might have a better outcome.

## Conclusions

A higher CAR was an independent risk factor for both initial and repeated IVIG resistance. Although it may predict both initial and repeated IVIG resistance in KD as a single parameter, its predictive ability was similar to ALB for initial IVIG resistance, as well as similar to ALB and CRP for repeated IVIG resistance. However, CAR might serve as a complementary laboratory marker regarding IVIG resistance prediction and provide some references for clinical management.

## Supplementary Information

**Additional file 1: Supplementary material 1** Ability of different scoring system to predict initial IVIG resistance in KD

**Additional file 2: Supplementary material 2.** Diagnostic specificity and sensitivity according to ROC-optimized decision limits for CAR, CRP, and ALB in predicting initial IVIG resistance among patients with KD

**Additional file 3: Supplementary material 3**. Ability of different scoring system to predict repeated IVIG non-responders in KD

**Additional file 4: Supplementary material 4.** Diagnostic specificity and sensitivity according to ROC-optimized decision limits for CAR, CRP, and ALB in predicting repeated IVIG non-responders among patients with KD

## Data Availability

All data generated or analyzed during this study are included in this published article and the supplementary files.

## Abbreviations

AHA: American heart association
ARP: Acute-phase proteins
ALB: Albumin
ALT: Alanine aminotransferase
AST: Aspartate aminotransferase
AUC: Area under the curve
BUN: Blood urea nitrogen
BSA: Body surface area
BMI: Body mass index
CBC: Complete blood count
CRP: C-reactive protein
CAR: C-reactive protein-to-albumin
CALs: Coronary artery lesions
ESR: Erythrocyte sedimentation rate
IVIG: Intravenous immunoglobulin
IL-1: Interleukin-1
IQR: Interquartile range
KD: Kawasaki disease
LAD: Left anterior descending artery
LCX: Left circumflex coronary artery
NPV: Negative predictive value
NLR: Neutrophil-to-lymphocyte ratio
STAMP: Screening tool for assessment of malnutrition in pediatrics
Na^+^: Sodium
ORs: Odds ratios
PLR: Platelet-to-lymphocyte ratio
PPV: Positive predictive value
ROC: Receiver operating characteristic
RCA: Right coronary artery
SD: Standard deviation
TBil: Total bilirubin
TNF: Tumor necrosis factor
WBC: White blood cell
WCSUH-SCU: West China second university hospital of Sichuan university
