# Supplementary material for: Predictive value of C-reactive protein to albumin ratio as a biomarker for initial and repeated intravenous immunoglobulin resistance in a large cohort of Kawasaki disease patients: a prospective cohort study
Source: Pediatr Rheumatol Online J. 2021 Mar 12;19:24. doi: 10.1186/s12969-021-00517-1 (PMC7953655; doi:10.1186/s12969-021-00517-1)
Supplement: Supplementary file 1 — Additional file 1: Supplementary material 1 Ability of different scoring system to predict initial IVIG resistance in KD [file 12969_2021_517_MOESM1_ESM.docx]

**Supplementary material 1** Ability of different scoring system to predict initial IVIG resistance in KD

|  | AUC | SE | 95%CI | *p* value | Cutoff points | Sensitivity | Specificity |
| --- | --- | --- | --- | --- | --- | --- | --- |
| **Model 1** | 0.599 | 0.029 | 0.556-0.640 | <0.0001 | 5.0 | 0.670 | 0.527 |
| **Model 2** | 0.642 | 0.030 | 0.600-0.682 | <0.0001 | 5.0 | 0.544 | 0.739 |

AUC, Area under the curve; CI, confidence interval; IVIG, intravenous immunoglobulin; KD, Kawasaki disease; SE, Standard Error;

Pairwise comparison of receiver operating characteristic (ROC) curves between model 1 and model 2 in predicting initial IVIG resistance by De Long test, *p*=0.170
