# Supplementary material for: Predictive value of C-reactive protein to albumin ratio as a biomarker for initial and repeated intravenous immunoglobulin resistance in a large cohort of Kawasaki disease patients: a prospective cohort study
Source: Pediatr Rheumatol Online J. 2021 Mar 12;19:24. doi: 10.1186/s12969-021-00517-1 (PMC7953655; doi:10.1186/s12969-021-00517-1)
Supplement: Supplementary file 2 — Additional file 2: Supplementary material 2. Diagnostic specificity and sensitivity according to ROC-optimized decision limits for CAR, CRP, and ALB in predicting initial IVIG resistance among patients with KD [file 12969_2021_517_MOESM2_ESM.docx]

**Supplementary material 2.** Diagnostic specificity and sensitivity according to ROC-optimized decision limits for CAR, CRP, and ALB in predicting initial IVIG resistance among patients with KD

|  | Estimated specificity at fixed sensitivity (n=550) | | | | Estimated sensitivity at fixed specificity (n=550) | | | |
| --- | --- | --- | --- | --- | --- | --- | --- | --- |
| CAR | Sensitivity(%) | Specificity(%) | Cut-off point | n | Specificity(%) | Sensitivity(%) | Cut-off point | n |
|  | 99.0 | 1.70 | 0.184 | 542 | 99.0 | 2.53 | 6.34 | 7 |
|  | 97.5 | 1.91 | 0.200 | 539 | 97.5 | 11.39 | 5.36 | 21 |
|  | 95.0 | 8.91 | 0.540 | 504 | 95.0 | 15.19 | 5.05 | 37 |
|  | 90.0 | 19.70 | 0.878 | 452 | 90.0 | 21.52 | 4.41 | 65 |
| CRP |  |  |  |  |  |  |  |  |
|  | 99.0 | 6.25 | 17.9 | 516 | 99.0 | 13.7 | 169.8 | 48 |
|  | 97.5 | 6.25 | 19.3 | 510 | 97.5 | 14.8 | 169.6 | 48 |
|  | 95.0 | 10.42 | 27.7 | 482 | 95.0 | 16.8 | 169.2 | 48 |
|  | 90.0 | 12.92 | 32.8 | 461 | 90.0 | 20.7 | 168.4 | 49 |
| ALB |  |  |  |  |  |  |  |  |
|  | 99.0 | 10.3 | 41.8 | 430 | 99.0 | 0.00 | 25.0 | 2 |
|  | 97.5 | 13.2 | 41.6 | 430 | 97.5 | 13.4 | 27.1 | 10 |
|  | 95.0 | 18.0 | 41.2 | 429 | 95.0 | 16.3 | 27.4 | 10 |
|  | 90.0 | 27.3 | 38.7 | 307 | 90.0 | 22.1 | 28.0 | 18 |

Abbreviations: ALB, albumin; CRP, C-reactive protein; CAR, C-reactive protein to albumin ratio; IVIG, intravenous immunoglobulin; KD, Kawasaki disease.
