# Supplementary material for: Predictive value of C-reactive protein to albumin ratio as a biomarker for initial and repeated intravenous immunoglobulin resistance in a large cohort of Kawasaki disease patients: a prospective cohort study
Source: Pediatr Rheumatol Online J. 2021 Mar 12;19:24. doi: 10.1186/s12969-021-00517-1 (PMC7953655; doi:10.1186/s12969-021-00517-1)
Supplement: Supplementary file 3 — Additional file 3: Supplementary material 3. Ability of different scoring system to predict repeated IVIG non-responders in KD [file 12969_2021_517_MOESM3_ESM.docx]

**Supplementary material 3**. Ability of different scoring system to predict repeated IVIG non-responders in KD

|  | AUC | SE | 95%CI | *p* value | Cutoff points | Sensitivity | Specificity |
| --- | --- | --- | --- | --- | --- | --- | --- |
| **Model 1** | 0.708 | 0.0534 | 0.595-0.805 | ＜0.001 | 3.5 | 0.645 | 0.771 |
| **Model 2** | 0.740 | 0.0532 | 0.629-0.832 | ＜0.001 | 3.0 | 0.774 | 0.646 |

AUC, Area under the curve; CI, confidence interval; IVIG, intravenous immunoglobulin; KD, Kawasaki disease; SE, Standard Error;

Pairwise comparison of receiver operating characteristic (ROC) curves between model 1 and model 2 in predicting repeated IVIG non-responders by De Long test, *p* = 0.629
