# Supplementary material for: Predictive value of C-reactive protein to albumin ratio as a biomarker for initial and repeated intravenous immunoglobulin resistance in a large cohort of Kawasaki disease patients: a prospective cohort study
Source: Pediatr Rheumatol Online J. 2021 Mar 12;19:24. doi: 10.1186/s12969-021-00517-1 (PMC7953655; doi:10.1186/s12969-021-00517-1)
Supplement: Supplementary file 4 — Additional file 4: Supplementary material 4. Diagnostic specificity and sensitivity according to ROC-optimized decision limits for CAR, CRP, and ALB in predicting repeated IVIG non-responders among patients with KD [file 12969_2021_517_MOESM4_ESM.docx]

**Supplementary material 4.** Diagnostic specificity and sensitivity according to ROC-optimized decision limits for CAR, CRP, and ALB in predicting repeated IVIG non-responders among patients with KD

|  | Estimated specificity at fixed sensitivity (n=79) | | | | Estimated sensitivity at fixed specificity (n=79) | | | |
| --- | --- | --- | --- | --- | --- | --- | --- | --- |
| CAR | Sensitivity(%) | Specificity(%) | Cut-off point | n | Specificity(%) | Sensitivity(%) | Cut-off point | n |
|  | 99.0 | 8.3 | 0.58 | 75 | 99.0 | 6.5 | 6.13 | 3 |
|  | 97.5 | 8.3 | 0.65 | 75 | 97.5 | 16.1 | 5.56 | 7 |
|  | 95.0 | 10.4 | 0.78 | 73 | 95.0 | 22.6 | 5.24 | 10 |
|  | 90.0 | 12.5 | 1.04 | 71 | 90.0 | 22.6 | 4.83 | 12 |
| CRP |  |  |  |  |  |  |  |  |
|  | 99.0 | 6.3 | 17.9 | 76 | 99.0 | 13.7 | 169.8 | 13 |
|  | 97.5 | 6.3 | 19.3 | 76 | 97.5 | 14.8 | 169.6 | 13 |
|  | 95.0 | 10.4 | 27.7 | 73 | 95.0 | 16.8 | 169.2 | 13 |
|  | 90.0 | 12.9 | 32.8 | 70 | 90.0 | 20.7 | 168.4 | 13 |
| ALB |  |  |  |  |  |  |  |  |
|  | 99.0 | 10.3 | 41.8 | 67 | 99.0 | 0.0 | 25 | 1 |
|  | 97.5 | 13.2 | 41.6 | 67 | 97.5 | 13.4 | 27.1 | 5 |
|  | 95.0 | 18.0 | 41.2 | 67 | 95.0 | 16.3 | 27.4 | 5 |
|  | 90.0 | 27.3 | 38.7 | 57 | 90.0 | 22.1 | 28.0 | 12 |

Abbreviations: ALB, albumin; CRP, C-reactive protein; CAR, C-reactive protein to albumin ratio; IVIG, intravenous immunoglobulin; KD, Kawasaki disease.
